# Eigenvector biomarker for prediction of epileptogenic zones and surgical success from interictal data

**DOI:** 10.3389/fnetp.2025.1565882

**Published:** 2025-05-20

**Authors:** Sayantika Roy, Armelle Varillas, Emily A. Pereira, Patrick Myers, Golnoosh Kamali, Kristin M. Gunnarsdottir, Nathan E. Crone, Adam G. Rouse, Jennifer J. Cheng, Michael J. Kinsman, Patrick Landazuri, Utku Uysal, Carol M. Ulloa, Nathaniel Cameron, Sara Inati, Kareem A. Zaghloul, Varina L. Boerwinkle, Sarah Wyckoff, Niravkumar Barot, Jorge González-Martínez, Joon Y. Kang, Sridevi V. Sarma

**Affiliations:** ^1^ University of Rochester School of Medicine and Dentistry, Rochester, NY, United States; ^2^ Department of Biomedical Engineering, Johns Hopkins University, Baltimore, MD, United States; ^3^ Institute for Computational Medicine, Johns Hopkins University, Baltimore, MD, United States; ^4^ Department of Electrical and Computer Engineering, Texas Tech University, Lubbock, TX, United States; ^5^ Department of Neurology, Johns Hopkins University, Baltimore, MD, United States; ^6^ Department of Neurosurgery, University of Kansas Medical Center, Kansas City, KS, United States; ^7^ Department of Neurology, University of Kansas Medical Center, Kansas City, KS, United States; ^8^ Surgical Neurology Branch, National Institute of Neurological Disorders and Stroke, National Institutes of Health, Bethesda, MD, United States; ^9^ Barrow Neurological Institute, Phoenix Children’s Hospital, Phoenix, AZ, United States; ^10^ Department of Neurology, Beth Israel Deaconess Medical Center, Boston, MA, United States; ^11^ Department of Neurosurgery, University of Pittsburgh, Pittsburgh, PA, United States

**Keywords:** epilepsy, network physiology, dynamical network models, interictal, epileptogenic zone (EZ)

## Abstract

**Introduction:**

More than 50 million people worldwide suffer from epilepsy. Approximately 30% of epileptic patients suffer from medically refractory epilepsy (MRE), which means that over 15 million people must seek extensive treatment. One such treatment involves surgical removal of the epileptogenic zone (EZ) of the brain. However, because there is no clinically validated biomarker of the EZ, surgical success rates vary between 30%–70%. The current standard for EZ localization often requires invasive monitoring of patients for several weeks in the hospital during which intracranial EEG (iEEG) data is captured. This process is time-consuming as the clinical team must wait for seizures and visually interpret the iEEG during these events. Hence, an iEEG biomarker that does not rely on seizure observations is desirable to improve EZ localization and surgical success rates. Recently, the source-sink index (SSI) was proposed as an interictal (between seizure) biomarker of the EZ, which captures regional interactions in the brain and in particular identifies the EZ as regions being inhibited (“sinks”) by neighbors (“sources”) when patients are not seizing. The SSI only requires 5-min snapshots of interictal iEEG recordings. However, one limitation of the SSI is that it is computed heuristically from the parameters of dynamical network models (DNMs).

**Methods:**

In this work, we propose a formal method for detecting sink regions from DNMs, which has a strong foundation in linear systems theory. In particular, the steady-state solution of the DNM highlights the sinks and is characterized by the leading eigenvector of the state-transition matrix of the DNM. To test this, we build patient-specific DNMs from interictal iEEG data collected from 65 patients treated across 6 centers. From each DNM, we compute the average leading eigenvectors and evaluate their potential as a biomarker to accurately predict EZ and surgical success.

**Results:**

Our findings show the ability of the leading eigenvector to accurately predict EZ (average accuracy 66.81% ± 0.19%) and surgical success (average accuracy 71.9% ± 0.22%) with data from 65 patients across 6 centers from 5 min of data, which we show is comparable with the current method of localizing the EZ over several weeks.

**Discussion:**

This eigenvector biomarker has the potential to assist clinicians in localizing the EZ quickly and thus increase surgical success in patients with MRE, resulting in an improvement in patient care and quality of life.

## Introduction

More than 50 million people worldwide suffer from epilepsy, a disorder characterized by repeated, unprovoked seizures in the brain due to abnormal electrical firing of neurons (World Health Organization, 2024). Approximately one-third of epileptic patients cannot be treated with medication and are subsequently diagnosed with medically refractory epilepsy (MRE) (Granata et al., 2009; Sinha et al., 2017; Gallagher et al., 2024). The most effective way to treat MRE is by surgically resecting the epileptogenic zone (EZ), which is the cortical region responsible for the generation and early spread of seizures (Lüders et al., 2006). The success of surgical outcomes varies often due to the inability to accurately locate the EZ. To localize the EZ, a patient may spend 2–3 weeks in an epilepsy monitoring unit in a hospital while their neural activity data is collected from intracranial electrodes (Bernabei et al., 2022; Bernabei et al., 2023; Sinha et al., 2023). Throughout the patient’s stay, the electrodes record activity both during seizures (ictal phase), and in between seizure events (interictal phase). The gold standard for identifying the EZ requires clinicians to spend many hours visually examining intracranial EEG (iEEG) recordings during seizure events to accurately pinpoint the EZ (Bernabei et al., 2022). Clinicians look for signatures of the EZ including low voltage fast activity (Bernabei et al., 2023; Litt et al., 2001). Despite large volumes of data collected from MRE patients, surgical success rates vary from 30% to 70% (Bernabei et al., 2023; Jobst and Cascino, 2015; González-Martínez et al., 2007; Malmgren and Edelvik, 2017; Bulacio et al., 2012; McIntosh et al., 2004). Such grim outcomes stem from reliance on capturing iEEG during seizure events and visual inspection of iEEG which is prone to human error and requires EEG expertise.

In this paper, we present a method to automatically identify the EZ from interictal iEEG data. Our approach has the potential to save time and money because clinicians could spend less time analyzing the data and more time treating more patients due to the short amount of interictal data needed. Furthermore, patients could spend less time in the epilepsy monitoring unit, which reduces time and risks associated with electrode implantation in the brain (Rosenow and Lüders, 2001).

The prevailing method for identifying the EZ from interictal data has been high-frequency oscillations (HFOs) analysis (Gliske et al., 2016; Nariai et al., 2019; Varatharajah et al., 2018; Murphy et al., 2017; Akiyama et al., 2011; Cimbalnik et al., 2019). HFOs have been well studied both in research and in clinical trials, but there are mixed results as to whether they are a reliable marker. HFOs are not well-defined and there is difficulty deciphering clinically important and naturally occurring HFOs (Gliske et al., 2018; Park and Hong, 2019). Detecting HFOs also consists of preprocessing the signals through methods, such as applying bandpass filters, that require signal processing knowledge.

More recently, the source‐sink index (SSI) was proposed as an interictal biomarker of the EZ, which captures regional interactions in the brain (Gunnarsdottir et al., 2022). The SSI outperformed HFO analysis when compared using iEEG recordings from 65 MRE patients from multiple centers (Gunnarsdottir et al., 2022). Patients with a successful outcome (Engel 1) were able to have their surgical success predicted, whereas patients with an unsuccessful outcome (Engel 2–4) could not be as accurately predicted using the SSI. In particular, the SSI identifies the EZ as regions in the brain acting as “sinks”, meaning they are inhibited by surrounding regions, referred to as “sources.” This characterization is based on interictal periods, when patients are between seizures. The SSI is derived from dynamical network models (DNMs) that are estimated from iEEG data. While the SSI performs well, it remains a heuristic measure derived from the DNM and is not directly grounded in systems theory. It is analogous to describing a road as “windy” versus providing the actual trajectory of the road.

In this study, we present a method that encapsulates the properties of the SSI by computing the leading eigenvectors of the state-transition matrices derived from the DNMs. These eigenvectors represent steady-state solutions, indicating the predicted trajectory of the multivariate iEEG signals as time approaches infinity. Moreover, our eigenvector-based approach can provide an explanation for the observed distinctions between regions of the brain associated with the EZ, and those that are not.

This work presents three main contributions. First, we present a biomarker grounded in dynamical systems theory for identifying the EZ. Second, we use this biomarker to accurately predict surgical success. Finally, we compare our results to the source sink index and find that our method does as well or better than the current methods for locating the EZ.

## Materials and methods

### Patient population

This retrospective study included 65 adults with medically refractory epilepsy, aged between 16 and 68 years (mean age 33.5 ± 13.0 years). The patients underwent intracranial EEG (iEEG) monitoring using stereotactically placed depth electrodes (stereo-EEG) and subsequently received surgical intervention. Post-iEEG treatments consisted of resective surgery (39 patients), laser ablation (17 patients), or responsive neurostimulation (9 patients; RNS). Patients were treated at one of six institutions: Cleveland Clinic (CC), Johns Hopkins Hospital (JHH), University of Kansas Medical Center (KUMC), University of Miami Hospital (UMH), National Institutes of Health (NIH), and University of Pittsburgh Medical Center (UPMC). All participants had at least 1 year of follow-up to assess treatment outcomes. A summary of patient demographics is provided in Table 1, with detailed clinical information available in Supplementary Table S1.

**TABLE 1:** Summary of patient information.

	CC	KUMC	JHU	NIH	UPMC	UMH	Total
Number of patients	29	9	5	9	5	8	65
Sex (male/female)	15/14	4/5	2/3	7/2	3/2	6/2	37/28
Age (years)	16–65	22–68	23–62	16–46	23–46	21–52	16–68
Surgical Outcome (success/failure)	13/16	4/5	0/5	4/5	3/2	1/7	28/37
MRI Findings (normal/abnormal)	26/3	6/3	0/5	5/4	4/1	5/3	46/19

### Stereo-EEG recordings

Stereo-EEG recordings are intracranial EEG (iEEG) obtained using EEG monitoring and diagnostic systems from Nihon Kohden or Natus (Natus Medical Inc.), with typical sampling rates of 1 or 2 kHz. A small portion of the data was collected at 500/512 Hz. Electrode placement was determined by the clinical team at each center. For analysis, one interictal snapshot was randomly selected per patient, with an average duration of 5.3 ± 4.2 min. Interictal periods were chosen at least one hour away from seizure events, without applying specific selection criteria, such as the presence or absence of epileptiform activity.

### Clinical annotations of the EZ

At each epilepsy center, the clinical team independently developed an EZ hypothesis for each patient as part of the presurgical evaluation, using both non-invasive scalp EEG and invasive iEEG data. The clinically annotated EZ refers to the anatomical region(s) targeted for treatment, whether through resection, ablation, or stimulation. This includes iEEG channels showing the earliest electrophysiological changes at seizure onset, commonly characterized by low-voltage fast activity (Litt et al., 2001). It is important to note that, since surgical treatment is guided by the EZ hypothesis (as well as early spread regions) with minor variations, there is typically significant overlap between the clinically annotated EZ and the areas ultimately treated for each patient.

### Data preprocessing

The iEEG data underwent bandpass filtering between 0.5 and 300 Hz using a fourth-order Butterworth filter, with notch filtering applied at 60 Hz and its harmonics (2 Hz stopband) to eliminate powerline interference. A common average reference was used to mitigate common noise across signals. Electrode locations were determined by combining co-registered post-implantation CT and brain MRI data, processed with tools such as BioImage Suite52, and subsequently validated by the clinical team at each center for accuracy. Channels not recording from gray matter (e.g., those in white matter or outside the brain) or identified as problematic (e.g., broken, excessively noisy, or containing artifacts) were excluded from the dataset. On average, 95 ± 32 iEEG channels per patient were retained for analysis. The sEEG data were segmented into non-overlapping 500 m windows for modeling and feature extraction (described in detail below). MATLAB R2020b (MathWorks, Natick, MA) was used for data processing and analysis, while Python 3.6+ (Python Software Foundation, Wilmington, DE) was employed for building models to predict surgical outcomes.

### Dynamical network models (DNMs)

Dynamical network models (DNMs) are a type of generative model designed to capture the dynamic interactions between individual iEEG channels within a network. The interictal DNM is represented as a linear time-varying (LTV) model, which mathematically describes the interactions between observed brain regions (iEEG channel signals) over time. The LTV model is constructed as a series of linear time-invariant (LTI) DNMs, each derived from smaller temporal segments of the data. The structure of each LTI model can be expressed as follows:

$$x\left(t+1\right)=Ax\left(t\right)+e\left(t\right).$$ (1)

In this context, N is the total number of channels, $x\left(t\right)\in R^{N}$ represents the iEEG channel signals, $A\in R^{N\times N}$ is the state transition matrix that characterizes the interactions and temporal evolution of the iEEG channels, and $e\left(t\right)\in R^{N}$ is white Gaussian noise independent of the initial measurements x(0) . A multivariate autoregressive (MVAR) model, commonly used to analyze effective connectivity in brain networks, adopts the form of an LTI system at each time lag. The LTI DNM can be viewed as a specific case of a first-order MVAR model, where interactions are considered one time step in the past. In previous work, we demonstrated that DNMs can be effectively constructed using least squares estimation and shown to accurately reconstruct iEEG signals (Gunnarsdottir et al., 2022).

### Leading eigenvectors versus sinks of DNMs

Systems theory provides a framework to analyze the dynamics and characteristics of DNMs, aiding in the precise localization of the EZ. Within these models shown in (Equation 1), the element $A_{ij}$ represents the influence of the current activity of channel j on the future activity of channel i . More broadly, the i -th row of A captures the cumulative functional effect of the network on channel i , while the j -th column reflects the influence that channel j exerts on the entire network. To compute the SSI, the norms of each row and column of A are computed as described in (Gunnarsdottir et al., 2022). In this paper, we show that the leading eigenvector of the A matrix can capture the strongest sinks in a network without computing a heuristic on its rows and columns. Instead, we use the leading eigenvector of the network as a biomarker for predicting surgical outcome and EZ.

**FIGURE 1:**
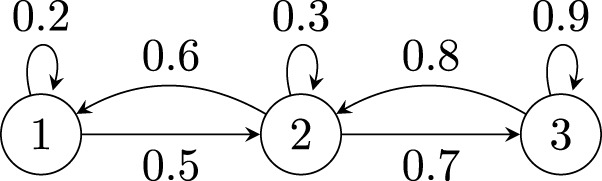
Illustrative three node network.

In this section, we will illustrate the computation and meaning behind the leading right eigenvector. To start, let’s consider a network of three nodes–see Figure 1.

The network has the following adjacency matrix

$$A=\left[\begin{array}{ccc} 0.2 & 0.6 & 0\\ 0.5 & 0.3 & 0.8\\ 0 & 0.7 & 0.9 \end{array}\right].$$ (2)

To find the right eigenvector of A in (Equation 2), we must find the eigenvalue decomposition, which determines the eigenvalues (energy) and eigenvectors (direction) of A . Suppose we consider the largest eigenvalue, which we will denote as $\lambda_{1}$ , and its corresponding eigenvector, which is a 3×1 vector denoted as $v_{1}$ , then

$$Av_{1}=v_{1}\lambda_{1}.$$ (3)

In Equation 3, $v_{1}$ is the leading right eigenvector for $\lambda_{1}$ . Simply stated, when the adjacency matrix A is multiplied with its right eigenvector $v_{1}$ , then the result is a scaled version of the right eigenvector, that is $\lambda_{1}$ scales $v_{1}$ .

Using our example network, when taking the eigenvalue decomposition, the largest eigenvalue is $\lambda_{1}=1.487$ , so the right eigenvector is given as

$$\underset{A}{\underbrace{\left[\begin{array}{ccc} 0.2 & 0.6 & 0\\ 0.5 & 0.3 & 0.8\\ 0 & 0.7 & 0.9 \end{array}\right]}}\underset{v_{1}}{\underbrace{\left[\begin{array}{c} 0.287\\ 0.6155\\ 0.734 \end{array}\right]}}=\underset{v_{1}}{\underbrace{\left[\begin{array}{c} 0.287\\ 0.6155\\ 0.734 \end{array}\right]}}\underset{\lambda_{1}}{\underbrace{1.487}}.$$ (4)

Note that the largest component in $v_{1}$ (Equation 4) is the third component corresponding to node 3. This also happens to be the largest “sink” [formerly defined in (Gunnarsdottir et al., 2022)] in the network in that there is more incoming influence to node 3. Similarly, node 1 is considered to be more of a “source” in this example because it has more outgoing influence.

To explain why the components of the leading eigenvector point to sinks in the network, we consider the steady-state solution of the system described in (Equation 1). For simplicity, we let e(t)=0 for all time. Let’s look at what happens to x(t) for a given initial condition x(0) over time.

$$x\left(1\right)=Ax\left(0\right),\;\;\;\;\;x\left(2\right)=Ax\left(1\right)=A^{2}x\left(0\right),\;\;\;\;\;\to \;\;\;x\left(t\right)=A^{t}x\left(0\right)=\overset{N}{\underset{i=1}{\sum }}{\left(\lambda_{i}\right)}^{t}v_{i}w_{i}^{T}x\left(0\right)$$ (5)

In Equation 5, $w_{i}$ is the i th left eigenvector of A . Now, note that $w_{i}^{T}x\left(0\right)$ is a scalar quantity, we call $\alpha_{i}\in R$ , so

$$x\left(t\right)=A^{t}x\left(0\right)=\overset{N}{\underset{i=1}{\sum }}{\left(\lambda_{i}\right)}^{t}\alpha_{i}v_{i}$$ (6)

Finally in Equation 6, without loss of generality, if we order and label the eigenvalues as $\lambda_{1}\gg \lambda_{2}\gg \lambda_{3}\gg \ldots \gg \lambda_{N}$ , then as t gets very large

$$x\left(t\right)\approx {\left(\lambda_{1}\right)}^{t}\alpha_{1}v_{1}$$ (7)

Equation 7 indicates that the steady-state solution x(t) points in the direction of the leading eigenvector (the eigenvector associated with the largest eigenvalue). In the context of sinks, the leading eigenvector can be seen intuitively as follows. Suppose $x_{i}\left(t\right)$ is the amount of water in bucket i , and at each time step, water gets poured from bucket j to bucket i according to $A_{ij}$ . Then after a long time, all the water will go into the buckets that are the sink nodes - hence the connection to the SSI.

Our hypothesis states that the values of the leading right eigenvector associated with the EZ channels will differ significantly from those of the non-EZ channels. This hypothesis is grounded in the premise that the steady-state solutions for the EZ and non-EZ channels, as expressed by the leading right eigenvector, exhibit inherent differences. The leading right eigenvector is of interest because it represents the steady-state solution of the linear time-invariant dynamics underlying the system. The Algorithm found in Box 1 outlines the procedure we followed to obtain the leading right eigenvectors from our data.

BOX 1 **| Our proposed novel biomarker feature.**

1:  **Input:** 5 minute snapshots of filtered and pre-processed interictal data
2:  **Output:** Our proposed novel biomarker feature
3:  Using least-squares, compute a series of A matrices across the data from 500 ms time windows
4:  The leading right eigenvector $v_{1}$ in Equation 3 is computed for all A matrices
5:  The average leading right eigenvector $v_{\mathrm{avg}}$ is computed across all time windows
6:  From the average leading right eigenvector $v_{\mathrm{avg}}$ , the elements associated with EZ channels are averaged to obtain a single value $v_{EZ}$ for all EZ channels
7:  From the average leading right eigenvector $v_{\mathrm{avg}}$ , the elements associated with non-EZ channels are averaged to obtain a single value $v_{\mathrm{nEZ}}$ for all non-EZ channels
8:  The final biomarker is computed by taking the difference between $v_{EZ}$ and $v_{\mathrm{nEZ}}$ , that is $\theta =v_{EZ}-v_{\mathrm{nEZ}}$

### Logistic regression models

To evaluate the predictive power of the leading eigenvector components (EVCs), we trained and tested three models using three sets of features: EVCs, source sink metrics (SSMs), and EVCs combined with SSMs. To predict clinically-annotated EZ (CA-EZ) versus non-EZ (CA-nEZ), we developed the following logistic regression models–see Table 2.

**TABLE 2:** Logistic Regression Models for Predicting EZ v. non-EZ.

	Name	Features
Model 1	EVC	average leading eigenvector
Model 2	SSMs	average sink index, average source influence, average sink connectivity
Model 3	Combined	average leading eigenvector, average sink index, average source influence, average sink connectivity

The logistic regression models in Table 2 were tested on the channels of only Engel 1 patients having accurately localized clinically annotated EZ to determine if the model could distinguish the channels within and outside of the EZ. 26 of the 28 Engel 1 patients were determined by clinician review to have an accurately localized EZ. For each model, the data was split into training and test sets, where the test set contained all of the channels from one patient. Thus, each model had 26 folds of cross validation. These models were fit to the training set and an ROC curve was generated. The optimal decision threshold was determined from training and then applied to the testing patient to predict EZ and non-EZ channels. Accuracy, sensitivity, and specificity of the predictions were calculated.

To predict successful (Engel 1) versus failure (Engels 2–4) surgical outcomes, we developed the following logistic regression models–see Table 3.

**TABLE 3:** Logistic regression models for predicting surgical outcome.

	Name	Features
Model 1	EVC	average difference in average leading eigenvector (EZ - non-EZ channels)
Model 2	SSMs	average difference in sink index, average difference in source influence, average difference in sink connectivity
Model 3	Combined	average difference in average leading eigenvector, average difference in sink index, average difference in source influence, average difference in sink connectivity

For each model in Table 3, we performed a 10-fold cross validation. These models were fit to the training set and an ROC curve was generated. The optimal decision threshold was determined from the training data and then applied to the testing fold to predict surgical outcome. Accuracy, sensitivity, and specificity of the predictions were calculated.

## Results

### The leading eigenvector correlates to the source sink index

**FIGURE 2:**
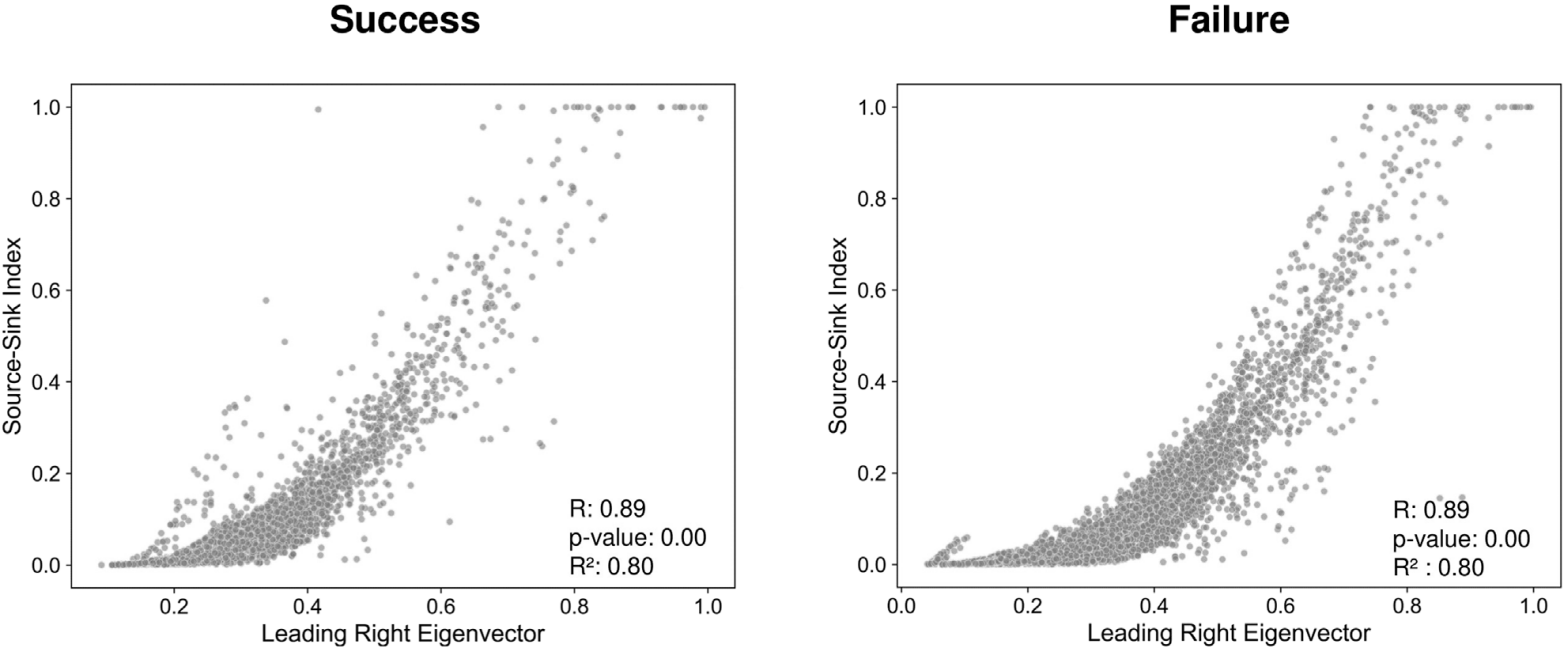
Source-Sink Index (SSI) versus Eigenvector Components (EVCs) across patients where success cases are considered to be Engel 1 and failure cases are considered to be Engel 2-4.

Figure 2 illustrates that the leading eigenvector components (EVCs), correlate to the source sink indices for each channel. The EVCs and source sink indices were averaged across time for each channel and patient. The data reveals a clear nonlinear dependency between the two variables, along with a statistically significant linear correlation (p<0.001) . Furthermore, the Supplementary Figure S1 demonstrates that the leading eigenvector components correlate to all source-sink metrics introduced in (Gunnarsdottir et al., 2022).

### The leading right eigenvector points to EZ channels in successful surgical outcomes

**FIGURE 3:**
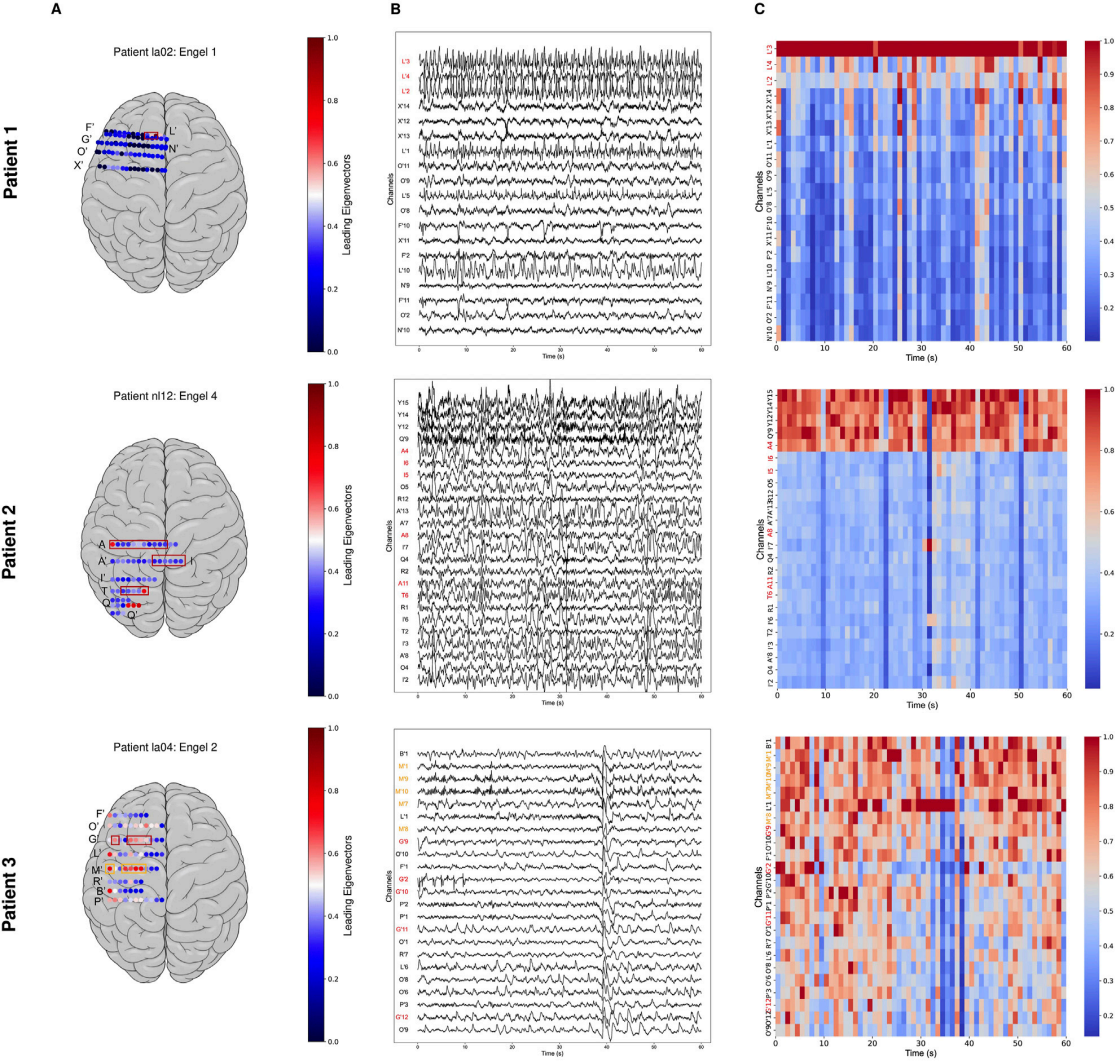
Three patient examples. Patient 1 had a successful surgical outcome (Engel 1). Patient 2 (middle) had a failed surgical outcome (Engel 4). Patient 3 (bottom) had two surgeries. After the first surgery, Patient 3 continued to have seizures (failed outcome) but became seizure-free (successful outcome) after the second surgery (Engel 2). **(A)** Average leading right eigenvector corresponding to each channel overlaid on a brain implantation map for each patient. CA-EZ is shown in a red box. **(B)** A 1-min interictal iEEG snapshot and the resulting leading eigenvector of every channel. Channels are arranged from highest to lowest value of the average leading eigenvector. CA-EZ channels are labeled in red text. For Patient 3, the CA-EZ from the second surgery is labeled in orange text. Only the top 30% of channels are shown for better visualization, and all channels not shown have low eigenvector component values (EVCs). **(C)** The resulting EVC of every channel. In Patient 1 (top), CA-EZ channels had the highest EVC values, whereas only 1 of 6 CA-EZ channels had high EVCs in Patient 2 (middle). In Patient 3 (bottom), the CA-EZ that rendered the patient seizure-free had the highest EVC values.

For each patient, we computed the leading right eigenvectors and the source sink indices from the DNMs. Figure 3 presents examples from three different patients (Engel 1, 2, and 4). For each patient, Figure 3 shows the implantation map for the placement of the electrodes, a sample snapshot of the iEEG, and the leading eigenvectors over the sample snapshot. As seen in Figure 3, the clinically annotated EZ (CA-EZ) has the largest eigenvector component values (EVCs) for all patients.

**FIGURE 4:**
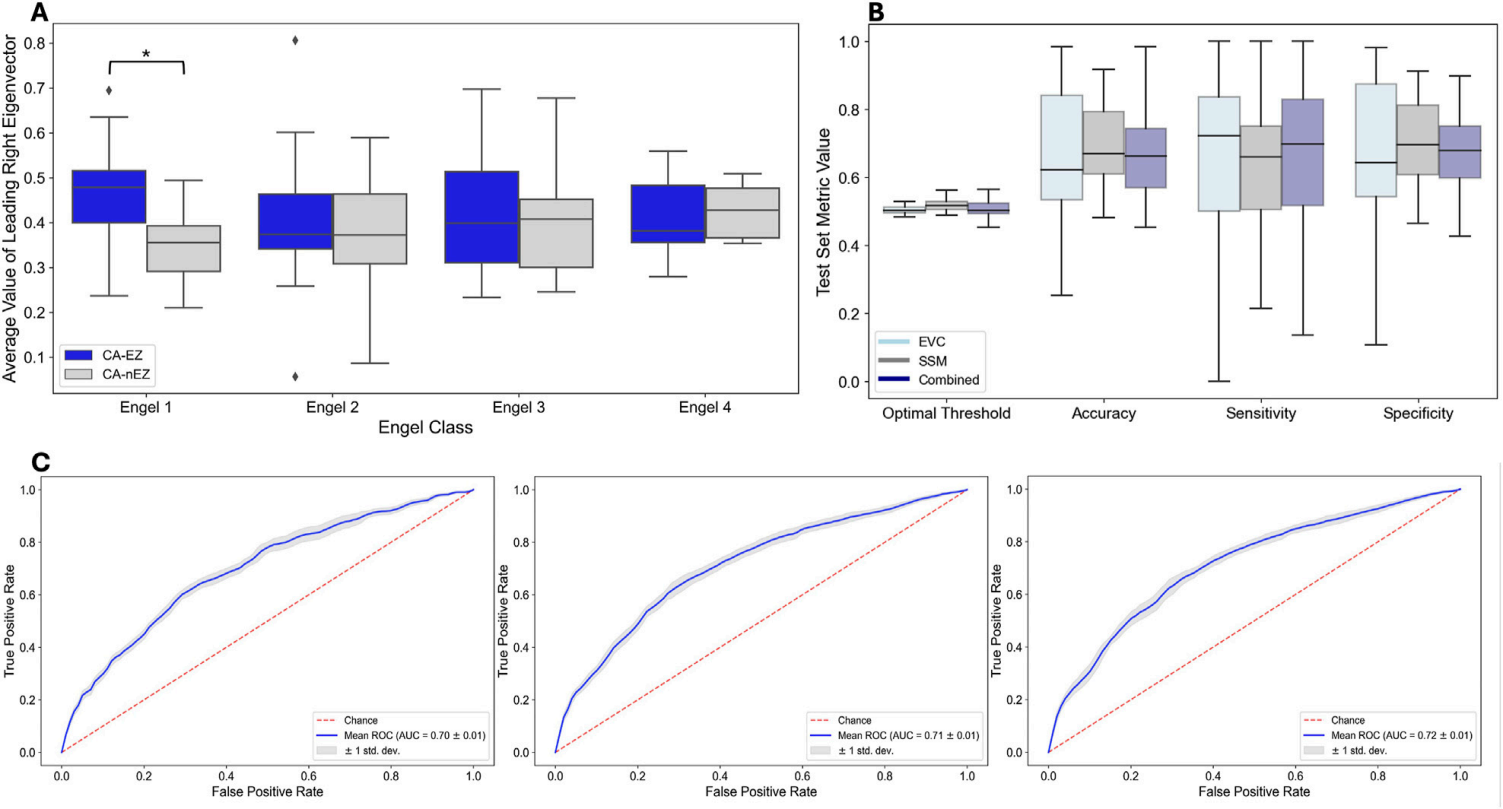
Predicting EZ vs. non-EZ Channels - Performance of Three Models: EVCs, SSM and Combined. **(A)** Distributions of leading eigenvector components for clinically annotated EZ and non-EZ across surgical outcomes. The diamond signifies outliers. **(B)** Distribution of performance metrics across test patients in each fold. **(C)** ROC curves on training data for each model.

We trained and tested three models (EVCs, SSMs, Combined) to predict EZ v. non-EZ. Figure 4A shows the distributions of leading eigenvector components for clinically annotated EZ and non-EZ across patients stratified by surgical outcomes, Engel 1–4. As shown in Figure 4A, the leading eigenvector components of the EZ are significantly higher ( p= 3.07 x $10^{-6}$ ) than those of non-EZ for Engel 1, but this is not the case for Engel 2–4. This suggests that there is a high correspondence between the clinically annotated EZ and the largest components of the leading eigenvector in patients who became seizure free after surgery. For Engel 1 patients, the EZ is assumed to be localized accurately. In Engel 2-4 outcomes, patients are not seizure free, and thus it is possible that the EZ was not accurately localized. In these patients, the channels with the largest eigenvector components do not all correspond to the EZ.

Figure 4B shows the distributions of the performance metrics across test patients for each of the three models: EVCs, SSMs, and combined. Note that all models perform comparably, which is not surprising as the EVCs are highly correlated to the SSMs–see Figure 2. The mean accuracy, sensitivity, and specificity for each of the models are shown in Table 4.

**TABLE 4:** Predicting EZ channels.

Model	Mean accuracy	Mean sensitivity	Mean specificity
EVC	66.81%±0.19%	62.53% ±0.30%	66.23% ±0.23%
SSM	68.57%±0.12%	60% ±0.28%	70.2%±0.12%
Combined	67.02% ±0.13%	63.46%±0.29%	67.91% ±0.13%

Bold values indicate the highest value of the performance metric (accuracy, sensitivity, and specificity, respectively) among the three models.

Figure 4C shows the ROC curves on the training data for each model. The mean AUC statistics across training folds for the EVC, SSM, Combined models are 0.70 ± 0.01, 0.71 ± 0.01, 0.72 ± 0.01, respectively.

### The leading eigenvector predicts surgical outcomes

**FIGURE 5:**
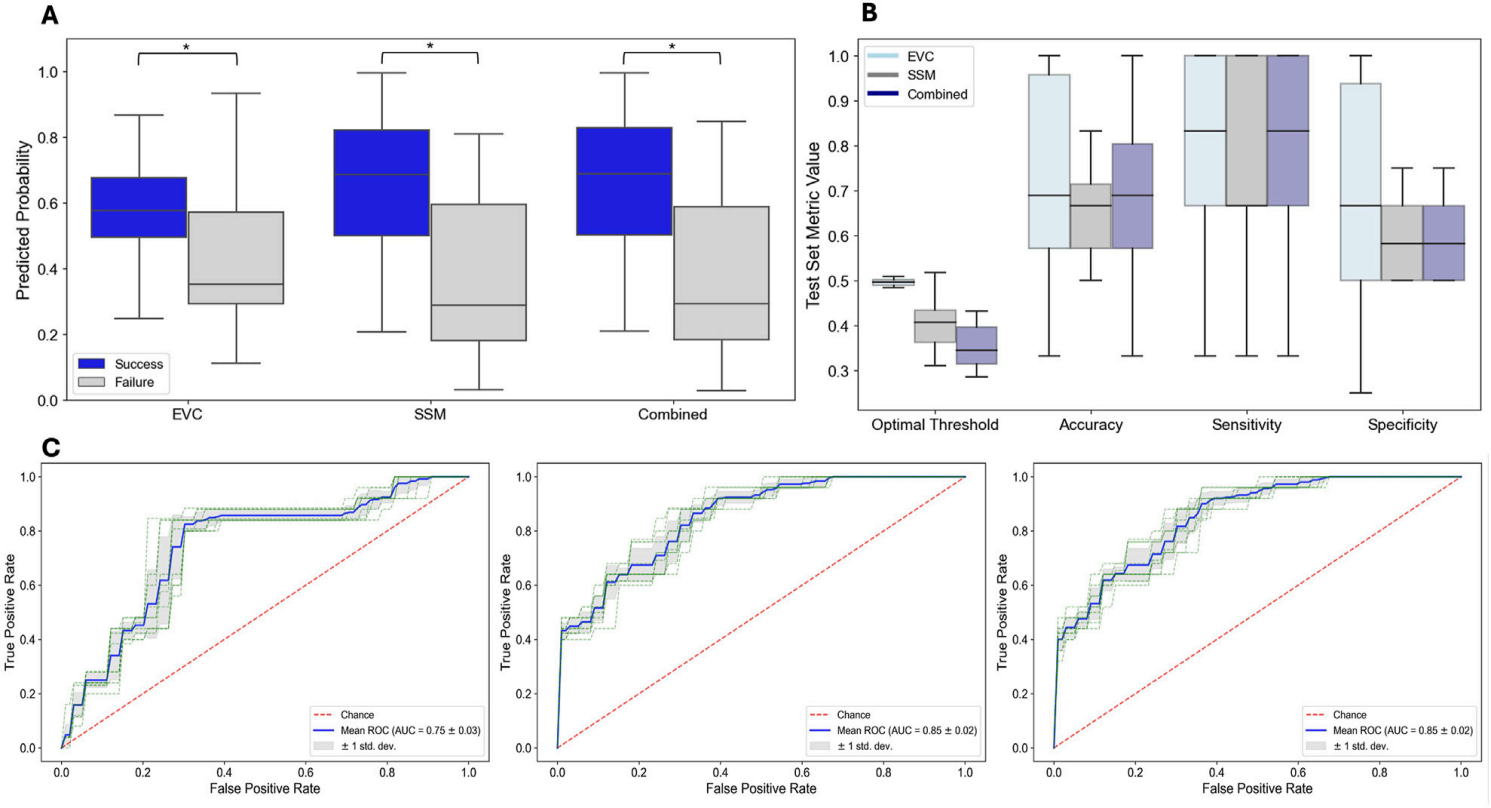
Predicting Surgical Outcome - Performance of Three Models: EVCs, SSM, and Combined. **(A)** Distribution of performance metrics across test patients in each fold. **(B)** Model predicted probabilities for each model and for each surgical outcome for test patients. **(C)** ROC curves on training data for each model.

Finally, we trained and tested three models (EVCs, SSMs, Combined) to predict surgical outcomes. Figure 5A shows the distributions of predicted probabilities for test patients for success (Engel 1) and failed surgical outcomes (Engels 2–4). As shown in Figure 5A, the predicted probabilities of each model are higher for success patients versus failed patients (EVC: p = 6.55 x $10^{-6}$ , SSM: p = 7.47 x $10^{-4}$ , Combined: p = 4.17 x $10^{-6}$ ). Again, these models suggest that there is a high correspondence between the clinically annotated EZ and the largest components of the leading eigenvector in patients who became seizure free after surgery. In Engel 2-4 outcomes, patients are not seizure free, and thus it is possible that the EZ was not accurately localized. In Engel 2-4 patients, the channels with the largest eigenvector components do not all correspond to the EZ.

Figure 5B shows the distributions of the performance metrics across test patients for each of the three models: EVCs, SSMs, and Combined for predicting surgical outcome. Note that all models perform comparably, which is again not surprising as the EVCs are highly correlated to the SSMs–see Figure 2. The mean accuracy, sensitivity and specificity for each of the models are shown in Table 5.

**TABLE 5:** Predicting surgical success.

Model	Mean accuracy	Mean sensitivity	Mean specificity
EVC	71.9%±0.22%	78.33%±0.24%	68.33%±0.24%
SSM	67.38% ±0.15%	75% ±0.23%	62.5% ±0.15%
Combined	67.38% ±0.18%	78.33%±0.24%	60% ±0.19%

Bold values indicate the highest value of the performance metric (accuracy, sensitivity, and specificity, respectively) among the three models.

Figure 5 shows the ROC curves on the training data for each model. The mean AUC statistics across training folds for the (EVC, SSM, Combined) models are 0.75 ± 0.03, 0.85 ± 0.02, 0.85 ± 0.02, respectively.

## Discussion

Previously, the source-sink metrics (SSMs) as interictal iEEG markers were proposed to support the localization of the epileptogenic zone (EZ) (Gunnarsdottir et al., 2022). These metrics are grounded in the hypothesis that seizures are suppressed when epileptogenic regions are effectively inhibited by neighboring areas. Our current study aimed to assess the effectiveness of the leading eigenvector components (EVCs) in capturing the SSMs into one theoretically sound measure. We tested the EVCs across a diverse patient cohort, encompassing various epilepsy etiologies, treatment approaches, and post-treatment outcomes. The iEEG data were sourced from 6 different clinical centers, resulting in a heterogeneous dataset that included patients with varying case complexities (e.g., lesional vs. non-lesional, and temporal vs. extra-temporal epilepsy), epilepsy types (focal and multi-focal), and clinical practices. This diversity reflects real-world conditions and aligns with the standard care success rates, averaging approximately 50%.

Among the 28 patients in our dataset with successful outcomes, the EVC interictal iEEG marker aligned with clinical assessments in 26 cases (93%). Conversely, for patients with unsuccessful outcomes, agreement with clinicians was observed in only 54% of cases. This indicates that the algorithm often identified additional potentially epileptogenic regions not targeted in treatment. Moreover, the EVCs demonstrated comparable predictive accuracy for surgical outcomes as did the SSMs. The EVCs correctly predicted outcomes in 72% of cases, surpassing the 67% accuracy achieved by the SSMs alone.

### Why EVCs may disagree with clinicians in patients with failed surgical outcomes

Surgical treatment for epilepsy may fail for a variety of reasons, and in complex cases, removing the EZ alone may not be sufficient to achieve seizure freedom. For example, in multifocal epilepsy, removing the primary focus might lead to the emergence of seizures from other regions that were not clinically evident before surgery. As a result, the EVC algorithm might partially or fully align with the treated areas, even in cases where surgical outcomes are unsuccessful.

Failure can also stem from incorrect or incomplete localization of the EZ, as well as incomplete treatment of these areas, which often leads to seizure recurrence. This is particularly likely in cases where the implanted electrodes do not adequately sample the true EZ, making it difficult, if not impossible, for both clinicians and algorithms to identify its full extent or widespread nature.

In some situations, a complete resection of the EZ is not feasible due to the risk of causing significant neurological deficits, especially if the EZ is located in the eloquent cortex. For these patients, palliative treatments such as responsive neurostimulation (RNS) or deep brain stimulation (DBS) are increasingly used as alternatives to resective surgery. While these approaches can effectively reduce seizure frequency, only a small percentage of patients achieve complete seizure control. Therefore, patients undergoing RNS or DBS may still have failed outcomes, even when the areas highlighted by the source-sink algorithm overlap with the clinically-assessed EZ.

### Limitations and future work

Validating any iEEG marker remains a significant challenge because the epileptogenic zone (EZ) is a theoretical construct that cannot be directly measured. Consequently, there is no definitive ground truth for its precise location. Instead, the closest approximation is achieved retrospectively, assuming the EZ was included in the treated regions if surgical intervention results in seizure freedom. While the presurgical EZ hypothesis and the treated areas may not always perfectly align, particularly in patients treated with responsive neurostimulation, we defined the clinically-annotated EZ (CA-EZ) based on the presurgical hypothesis rather than the treated regions for two key reasons.

First, postoperative MRI data were not consistently available for research purposes across all centers, limiting our ability to confirm the exact locations of treated areas in some cases. Second, the EVC algorithm is intended as an assistive computational tool to aid clinicians in forming their EZ localization hypothesis. The goal is for the tool to complement existing clinical data, providing an additional layer of information for refining the EZ hypothesis. Despite some variability, the CA-EZ and the treated regions typically overlap significantly, as surgical planning is primarily based on the CA-EZ. Thus, we do not anticipate that the method of defining the EZ introduces bias into the metrics used to evaluate the algorithm’s performance.

Another limitation of this study is the reliance on 1-year post surgical outcomes. While achieving 1 year of seizure freedom holds some predictive value for long-term post-surgical outcomes, some patients may experience recurrence, leading to fewer individuals remaining in Engel Class 1 over time. Although this consideration extends beyond the scope of the current analysis, it raises curiosity about the robustness of the examined measures in relation to long-term outcomes, an aspect that will be explored in future research. Our study also lacks annotations of whether the interictal snapshots were captured during sleep or wake. Results may differ in different sleep stages when compared to wake and will be explored in future work.

Due to the spatial resolution limitations of iEEG contacts, the dynamical network models (DNMs) cannot differentiate between excitatory and inhibitory connections. The models only provide information about the degree of influence between network nodes. However, the strong predictive performance of the EVCs and SSMs suggest that the identified sources are predominantly influenced by inhibitory activity, aligning with the source-sink hypothesis.

Future research could aim to distinguish excitatory from inhibitory influences by combining iEEG with other modalities, such as resting-state functional MRI (rs-fMRI). While rs-fMRI has lower temporal resolution compared to iEEG, it generally offers higher spatial resolution, potentially enabling a more detailed understanding of the directionality and nature of network connections. Integrating these modalities could significantly enhance insights into the mechanisms underlying epileptic networks.

## Data Availability

The raw data supporting the conclusions of this article will be made available by the authors, without undue reservation. Requests to access these datasets should be directed to Sridevi V. Sarma ssarma2@jhu.edu. The code that was used in this data algorithm and processing can be accessed at: https://github.com/skroy576/eigenvectorbiomarker.
