# Supplementary material for: Eigenvector biomarker for prediction of epileptogenic zones and surgical success from interictal data
Source: Front Netw Physiol. 2025 May 20;5:1565882. doi: 10.3389/fnetp.2025.1565882 (PMC12129916; doi:10.3389/fnetp.2025.1565882)
Supplement: Supplementary file 1 [file DataSheet1.pdf]

Supplementary Material Eigenvector Biomarker for Predicting EZ and Surgical Success from Interictal Data

Sayantika Roy[1], Armelle Varillas[2,3], Emily A. Pereira[4], Patrick Myers[2,3], Golnoosh Kamali[3], Kristin Gunnarsdottir[2,3], Nathan E. Crone[5], Adam G. Rouse[6], Jennifer J. Cheng[6], Michael J. Kinsman[6], Patrick Landazuri[6], Utku Uysal[7], Carol M. Ulloa[7], Nathaniel Cameron[6], Sara Inati[8], Kareem A. Zaghloul[8], Varina L. Boerwinkle[9], Sarah Wyckoff[9], Niravkumar Barot[10], Jorge A. González-Martínez[11], Joon Y. Kang[*5], and Sridevi V. Sarma[*2,3]

[1]University of Rochester School of Medicine and Dentistry, Rochester, NY
[2]Department of Biomedical Engineering, Johns Hopkins University, Baltimore, MD
[3]Institute for Computational Medicine, Johns Hopkins University, Baltimore, MD
[4]Department of Electrical and Computer Engineering, Texas Tech University, Lubbock, TX
[5]Department of Neurology, Johns Hopkins University, Baltimore, MD
[6]Department of Neurosurgery, University of Kansas Medical Center, Kansas City, KS
[7]Department of Neurology, University of Kansas Medical Center, Kansas City, KS
[8]Surgical Neurology Branch, National Institute of Neurological Disorders and Stroke, National Institutes of Health, Bethesda, MD
[9]Barrow Neurological Institute, Phoenix Children's Hospital, Phoenix, AZ
[10]Department of Neurology, University of Pittsburgh, Pittsburgh, PA
[11]Department of Neurosurgery, University of Pittsburgh, Pittsburgh, PA

Patient Data

A detailed table summarizes the patient data used in this study – see Table 1.

Methods

In this work, we consider linear time-invariant dynamical network models, given as

$$x(t+1) = Ax(t), \tag{1}$$

where $x(t) \in \mathbb{R}^{n\times 1}$ is the state or measurement, $A \in \mathbb{R}^{n\times n}$ can be thought of as the adjacency matrix of the network, and $t \in \{1, 2, 3, \ldots\}$ is the time step.

The well-known solution to the linear time-invariant dynamical network model is given as

$$x(t) = A^t x(0), \tag{2}$$

where $x(0)$ are the initial conditions of the system.

If the matrix A is diagonalizable, then the eigenvalue decomposition of the solution is given as

$$x(t) = V\Lambda^t V^{-1} x(0), \tag{3}$$

where $V \in \mathbb{R}^{n\times n}$ is matrix whose columns are the eigenvectors of A and $\Lambda \in \mathbb{R}^{n\times n}$ is a diagonal matrix whose diagonal entries are the eigenvalues of A. Suppose we let $c = V^{-1}x(0)$, where $c \in \mathbb{R}^{n\times 1}$, then

$$x(t) = V\Lambda^t c. \tag{4}$$

Table 1: Detailed Summary of Patient Data

Patient ID	Center	Engel	Outcome	Race	Age	Hand	Gender	Surgery Type	Treated Contacts	EZ Contacts	MTLE/NMTLE	Epilepsy Type	MRI
**la02**	cc	1	S	0	19	R	F	ablation	L'1-5	L'2-4	NMTLE	Focal	Normal
**la03**	cc	2	F	0	37	R	M	ablation	L5-9	L7, W12-14, X6-10, O14-16, F10-12	NMTLE	Focal	Abnormal
**la04**	cc	2	F	0	32	R	M	ablation	L'2-6, G'1-3	L'4, G'1, g'2-15	NMTLE	Focal	Normal
**la05**	cc	2	F	0	29	R	M	ablation	T'1-4, D'1-4	D'1-2, U'3-5, D1-2, S'7-11, S7-11, T'1-3, T1-3	NMTLE	Focal	Normal
**la06**	cc	2	F	0	37	R	F	ablation	Q'1-6, R'1-6	Q'3-4; R'3-4	NMTLE	Focal	Normal
**la08**	cc	3	F	0	24	R	F	ablation	Q1-4	Q1-3, Q2	NMTLE	Focal	Normal
**la09**	cc	2	F	0	34	R	F	ablation	P'1-4	x'1-4, u'1-2, p'1-2	NMTLE	Focal	Normal
**la10**	cc	2	F	0	20	R	M	ablation	S1-4, R1-5	S1-2, R2-3	NMTLE	Focal	Normal
**la11**	cc	2	F	0	22	R	M	ablation	D4-8, Z8-12	D6, Z10	NMTLE	Focal	Normal
**la13**	cc	1	S	0	43	R	M	ablation	Y11-16	Y13-14	NMTLE	Focal	Normal
**la15**	cc	4	F	0	22	R	M	ablation	R1-5	R1-3	NMTLE	Multi-Focal	Normal
**la16**	cc	4	F	0	37	R	M	ablation	Q7-8	Y1-2, M'1-2, X'1-2, Q7-8	NMTLE	Multi-Focal	Normal
**la21**	cc	1	S	0	40	R	M	ablation	J6-9; X5-8	J6-9, X5-8, Y11-Y16	NMTLE	Focal	Abnormal
**la22**	cc	2	F	4	44	R	F	ablation	P3-4	P3-4	NMTLE	Focal	Normal
**la23**	cc	1	S	0	28	L	F	ablation	L1-2; O1-2; V1-2	L1-2, O1-2, V1-2, V4-5, C1-4, U1-6, F6-7, I1-10	NMTLE	Focal	Abnormal
**la24**	cc	2	F	0	19	R	F	ablation	C'6-7; L'6-7; O'6-7	C'6-7, L'6-7, O'6-7	NMTLE	Focal	Abnormal
**nl01**	cc	3	F	0	24	R	F	resection	M'8-10	M'8-12	NMTLE	Focal	Normal
**nl04**	cc	1	S	N/A	16	L	M	resection	R'1-6, H'5-10, S'6-10	R'1-5, Q'2-3, R6-7, Q1-2	NMTLE	Focal	Normal
**nl05**	cc	1	S	0	23	L	M	resection	W'1-12 , Y'1-14 , Z'1-9	M'7-9, W'2-3, W'9, W'10-12, Y'1-7, Y'10-13, Z'1-3, Z'9	NMTLE	Focal	Normal
**nl07**	cc	1	S	0	37	R	M	resection	R5-10	R5-7, Q7-9, R'5-7, m2-5	NMTLE	Focal	Normal
**nl08**	cc	1	S	0	16	R	F	resection	A'1-14, E'1-14, I'1-10	I'1-4, A'1-4, B'1-4, C'1-4, E'1-4	MTLE	Focal	Normal
**nl12**	cc	4	F	0	16	R	M	resection	A2-14; I1-8; T3-9	A2-14; I1-8; T3-9	NMTLE	Focal	Normal
**nl13**	cc	1	S	0	65	R	F	resection	B'1-4, C'1-3, T'1-10	B'1-4, C'1-3, T'1-9	NMTLE	Focal	Normal
**nl14**	cc	2	F	0	43	R	M	resection	B1-14; E1-12; F1-11, X1-16	X1-3, X5-6, X9-14, E4-5	NMTLE	Focal	Normal
**nl15**	cc	1	S	0	31	R	F	resection	A1-10; B1-10; I1-6; E1-7	B1-3, A1-3	MTLE	Focal	Normal
**nl16**	cc	1	S	0	25	R	F	resection	A'1-14; B'1-3; E'1-10; I'1-9	B'8-9, C'9-10, E'7-8, I'1-16	NMTLE	Focal	Normal
**nl18**	cc	1	S	0	18	R	F	resection	A'1-10; I1-8; T1-8; B1-14; C1-14	A1-2, B1-2, C1-4	NMTLE	Focal	Normal
**nl19**	cc	3	F	1	25	L	M	resection	A1-12; I1-9; T1-8	A1-2	NMTLE	Focal	Normal
**nl20**	cc	1	S	0	58	R	F	resection	A'1-10; I'1-7; T'4-6, B'1-10	A'1-10; I'1-7; T'4-6, B'1-10	MTLE	Focal	Normal
**pt1**	kumc	1	S	N/A	24	N/A	F	resection	RST5-8	RST5-8	NMTLE	Focal	Abnormal
**pt3**	kumc	1	S	N/A	35	N/A	F	resection	RTP1-6, RHH1-8	RHH1-2	MTLE	Multi-Focal	Abnormal
**pt4**	kumc	2	F	N/A	31	N/A	F	resection	LMC1-2	LAC1-2, LMC1	NMTLE	Focal	Normal
**pt6**	kumc	3	F	N/A	57	N/A	M	ablation	LHH1-3, LHB1-3	LHB1-3, LHH1-3	MTLE	Focal	Normal
**pt8**	kumc	2	F	N/A	27	N/A	M	resection	RHH1-14	RHH1-7	MTLE	Focal	Normal
**pt10**	kumc	1	S	N/A	35	N/A	F	resection	RAM1-6, RHB1-5	RHB1-4	MTLE	Focal	Normal
**pt11**	kumc	2	F	N/A	68	N/A	M	resection	LAM1-14, LHH1-14, LENC1-10	LSTG2, LSTGC1-2, LAM1-4, LHH1-3	MTLE	Multi-Focal	Normal
**pt12**	kumc	2	F	N/A	22	N/A	M	resection	OF7-9, LA3-6, MA1-6, MB1-6	OF8-9	NMTLE	Focal	Abnormal
**pt13**	kumc	1	S	N/A	58	N/A	F	resection	RHH1-8, RHB1-8, RAM1-8	RHH5-6	MTLE	Focal	Normal
**PY18N002**	jhu	2	F	N/A	62	N/A	M	resection	RHD1-2, RAD1-2	RHD1-2, RAD1-2, LMFD1-2, LOFD1-2	MTLE	Multi-Focal	Abnormal
**PY18N013**	jhu	1	S	N/A	24	N/A	F	resection	LTP1-4, LSTA1-2, LMTA2, L Amygdala	LTP1-4, LSTA1-2, LMTA2	MTLE	Focal	Abnormal
**PY18N015**	jhu	1	S	N/A	N/A	N/A	F	resection	LOF1-14	LOF1, LA1-3, LH1-3	NMTLE	Focal	Abnormal
**PY19N015**	jhu	3	F	N/A	23	N/A	F	RNS	N/A	LBT1-3, LHP1-2, LA1-2, LHA1-3	MTLE	Focal	Abnormal
**PY19N023**	jhu	1	S	N/A	32	N/A	M	resection	N/A	RA1-2, RAH1-2, RPH1-2, RMI3-5, RMM5-6	MTLE	Focal	Abnormal
**nih2**	nih	1	S	1	31	R	M	resection	RATO1-10; RPTO1-10; RPHD1-4; RIPI1-2; RAID1-2	RAID1-4, RIPI1-2, RPHD1-3	NMTLE	Multi-Focal	Normal
**nih3**	nih	1	S	0	36	R	F	resection	LAD1-12	LAD2-6, LAHD4-5, LAHD13-14, LAD10-12, LOF9-10, LPFC1-2	NMTLE	Multi-Focal	Normal
**nih4**	nih	1	S	0	39	R	M	resection	RSLP5-10, RILP5-10	RSLP8-9, RILP7, RSMP3	NMTLE	Multi-Focal	Abnormal
**nih5**	nih	1	S	0	41	R	M	resection	IEAF9-10, IAF8-11, SMF6-9, SPF7-8, SAF6-9	SAF6-7, IEPF3, SPF8-12, IPF9-11, SMF7-8, IEAF9-10	NMTLE	Focal	Normal
**nih7**	nih	3	F	0	46	R	M	resection	LAH1-6, LPH1-4, LALT6	LPH1-3, LAH1-5	MTLE	Multi-Focal	Abnormal
**nih8**	nih	2	F	0	37	R	M	resection	LPT1-16	LPT1-6	MTLE	Focal	Normal
**nih9**	nih	3	F	0	16	L	F	resection	PLF1-3, ALP1-4, PLP1-6	AID4-8	NMTLE	Focal	Abnormal
**nih10**	nih	2	F	0	25	R	M	resection	LAID2-10	LAID6-8	NMTLE	Focal	Normal
**nih11**	nih	2	F	0	27	R	M	resection	MAF4-10, LSA4-8, LMA4-8, LIA7-8	LSA5-8, MPF6-12, LIA1-6	NMTLE	Focal	Abnormal
**upmc1**	upmc	1	S	N/A	44	R	M	resection	A1-16, B1-16, C1-5, T1-16, I1-16, E1-16	B1-4, C3-5, A1-3	MTLE	Focal	Normal
**upmc2**	upmc	1	S	N/A	46	R	F	resection	A'1-16, B'1-16, C'1-5, T'1-16, I'-16, E'1-16	A'1-4, B'1-6, C'1-6, E'1-6	MTLE	Focal	Abnormal
**upmc3**	upmc	2	F	N/A	24	R	M	resection	A1-5, B1-5, E1-5, I1-5	A1-5, B1-5, E1-5	MTLE	Focal	Normal
**upmc5**	upmc	4	F	N/A	46	R	F	resection	J'10-15, I'1-16, A'1-16, E'1-16, B'1-16, T'3-8, C'1-4, F'1-10	T'3-6, E'1-5, F'2-4	NMTLE	Focal	Normal
**upmc6**	upmc	1	S	N/A	23	R	M	resection	J10-15, I1-16, A1-16, E1-16, B1-16, T3-8, C1-4	B1-4, A1-4, C1-4	MTLE	Focal	Normal
**002**	miami	3	F	N/A	36	R	F	RNS	N/A	DYS5-8	MTLE	Multi-Focal	Abnormal
**003**	miami	3	F	N/A	21	L	M	RNS	N/A	LIS1-6	NMTLE	Multi-Focal	Abnormal
**004**	miami	4	F	N/A	52	L	M	RNS	N/A	H1-2	MTLE	Focal	Normal
**006**	miami	1	S	N/A	49	R	M	RNS	N/A	LAI1-3, LSF3-4, LAC5-7, LMF5-7	NMTLE	Focal	Abnormal
**009**	miami	3	F	N/A	48	R	M	RNS	N/A	RTL1-3, RAT1-3	MTLE	Multi-Focal	Normal
**011**	miami	4	F	N/A	24	R	F	RNS	N/A	ROF6-12	NMTLE	Focal	Normal
**013**	miami	2	F	N/A	25	R	M	RNS	N/A	RAI9-12	MTLE	Focal	Normal
**015**	miami	2	F	N/A	27	R/L	M	RNS	N/A	LFP1-6	NMTLE	Multi-Focal	Normal

Since Λ is a diagonal matrix, then

$$\Lambda^t c = \begin{bmatrix} \lambda_1^t c_1 \\ \lambda_2^t c_2 \\ \vdots \\ \lambda_n^t c_n \end{bmatrix}. \tag{5}$$

By substituting, we obtain

$$x(t) = \begin{bmatrix} | & | & & | \\ v_1 & v_2 & \dots & v_n \\ | & | & & | \end{bmatrix} \begin{bmatrix} \lambda_1^t c_1 \\ \lambda_2^t c_2 \\ \vdots \\ \lambda_n^t c_n \end{bmatrix}. \tag{6}$$

By matrix multiplication in linear algebra, we obtain the following equivalent form

$$x(t) = v_1 \lambda_1^t c_1 + v_2 \lambda_2^t c_2 + \dots + v_n \lambda_n^t c_n = \sum_{i=1}^{n} v_i \lambda_i^t c_i, \tag{7}$$

where $c_i \in \mathbb{R}$ is a constant determined from the initial conditions, $v_i \in \mathbb{R}^n$ is an eigenvector, and $\lambda_i \in \mathbb{R}$ is its corresponding eigenvalue. If we order the summation from largest eigenvalue to smallest eigenvalue, then

we obtain

$$x(t) = v_{\max}\lambda_{\max}^t c_{\max} + \ldots + v_{\min}\lambda_{\min}^t c_{\min}. \tag{8}$$

Hence, the solution is approximately equal to the dominating term, which has the largest eigenvalue.

$$x(t) \approx v_{\max}\lambda_{\max}^t c_{\max} \tag{9}$$

If we were to compute the steady-state solution, then we would obtain

$$\lim_{t\to\infty} x(t) \approx \lim_{t\to\infty} v_{\max}\lambda_{\max}^t c_{\max}, \tag{10}$$

where we observe that the steady-state solution is dominated by the largest eigenvalue and corresponding eigenvector, denoted as the leading right eigenvector.

Our new characteristic biomarker is computed from the leading eigenvector.

Results

Figure 1 shows the correlation between EVCs and SSMs.

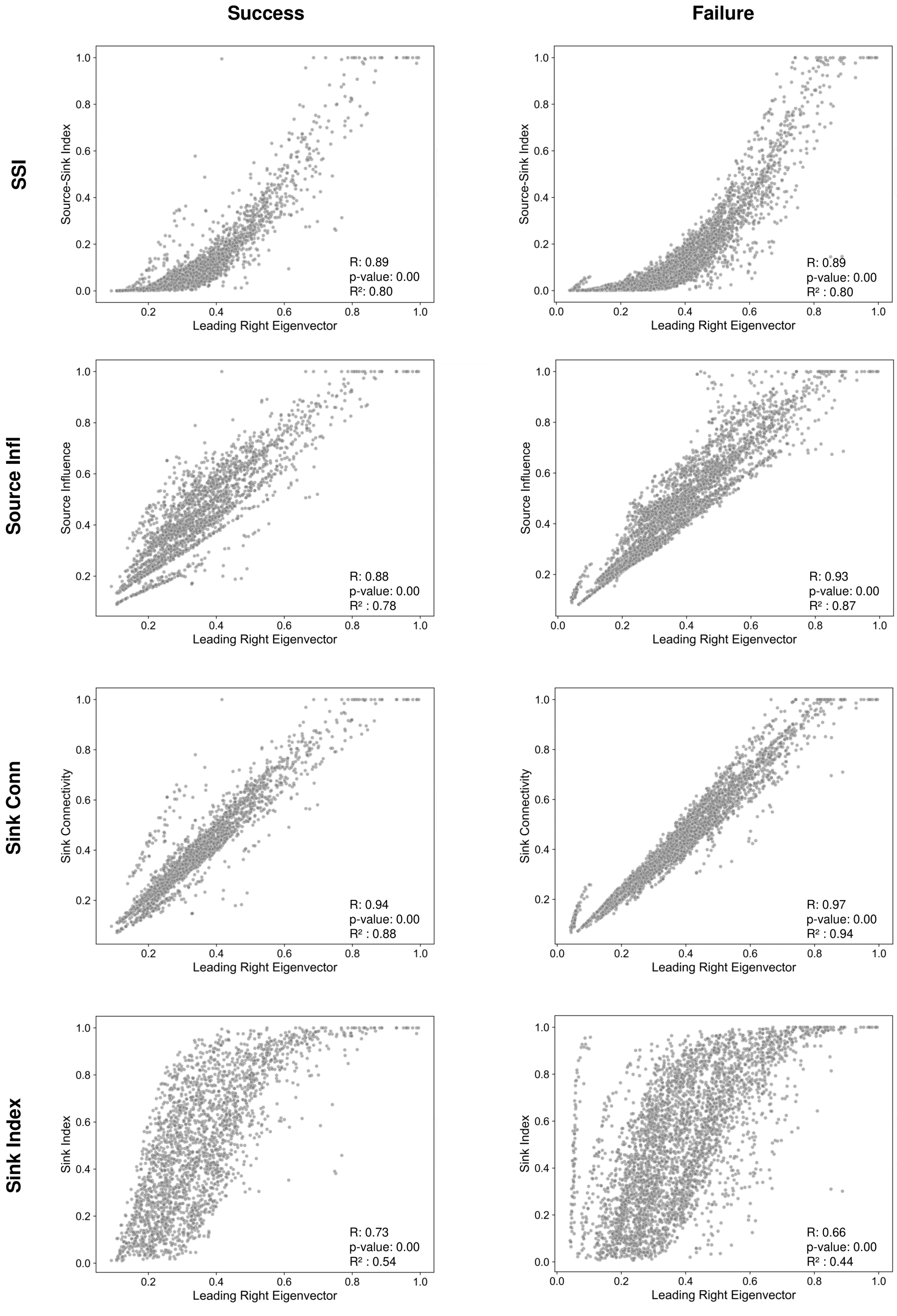

Figure 1: Eigenvector Components (EVCs) versus Source-Sink Indices, Source Influence, Sink Connectivity, and Sink Indices Across Patients
